# Surface Optimization and Design Adaptation toward Spheroid Formation On-Chip

**DOI:** 10.3390/s22093191

**Published:** 2022-04-21

**Authors:** Neda Azizipour, Rahi Avazpour, Mohamad Sawan, Abdellah Ajji, Derek H. Rosenzweig

**Affiliations:** 1 Institut de Génie Biomédical, Polytechnique Montréal, Montréal, QC H3C 3A7, Canada; neda.azizipour@polymtl.ca (N.A.); sawan@westlake.edu.cn (M.S.); 2 Department of Chemical Engineering, Polytechnique Montréal, Montréal, QC H3C 3A7, Canada; info@recutex.ca; 3 Polystim Neurotech Laboratory, Electrical Engineering Department, Polytechnique Montréal, Montréal, QC H3T 1J4, Canada; 4 CenBRAIN Laboratory, Westlake Institute for Advanced Study, School of Engineering, Westlake University, Hangzhou 310024, China; 5 The Research Center for High Performance Polymer and Composite Systems, Chemical Engineering Department, Polytechnique Montréal, Montréal, QC H3C 3A7, Canada; 6 Department of Surgery, McGill University, Montréal, QC H3G 1A4, Canada; 7 Injury, Repair and Recovery Program, Research Institute of McGill University Health Centre, Montréal, QC H3H 2R9, Canada

**Keywords:** microfluidic, 3D cell culture, spheroid, surface modification, PDMS, cancer

## Abstract

Spheroids have become an essential tool in preclinical cancer research. The uniformity of spheroids is a critical parameter in drug test results. Spheroids form by self-assembly of cells. Hence, the control of homogeneity of spheroids in terms of size, shape, and density is challenging. We developed surface-optimized polydimethylsiloxane (PDMS) biochip platforms for uniform spheroid formation on-chip. These biochips were surface modified with 10% bovine serum albumin (BSA) to effectively suppress cell adhesion on the PDMS surface. These surface-optimized platforms facilitate cell self-aggregations to produce homogenous non-scaffold-based spheroids. We produced uniform spheroids on these biochips using six different established human cell lines and a co-culture model. Here, we observe that the concentration of the BSA is important in blocking cell adhesion to the PDMS surfaces. Biochips treated with 3% BSA demonstrated cell repellent properties similar to the bare PDMS surfaces. This work highlights the importance of surface modification on spheroid production on PDMS-based microfluidic devices.

## 1. Introduction

Cancer is among the top causes of death in developed countries. According to the statistics in Canada [1], cancer causes over 30% of the total deaths in Canada annually. Major issues for successfully treating cancer include early diagnosis and proper radio-, chemo-, and/or surgical therapy. Chemotherapy has long stood as the adjuvant for radiation and surgical approaches; however, not all cancer types or patients respond to chemotherapy. One reason for this can be linked-to methods used for developing the chemotherapies, which rely heavily on in vitro two-dimensional cell culture and animal models which do not recapitulate the physiological human tumor microenvironment. Spheroids, tumoroids, and tumorospheres have been developed as compact and spherical three-dimensional (3D) cell agglomerations widely used in advanced preclinical oncology in vitro models [2,3,4]. Hence, loose cell aggregates that easily detach or other forms of spatial cell aggregations should not be considered spheroids, since the compactness and the spherical geometry of spheroids are the essential characteristics giving them in vivo-like tumor features to make them more reliable tools in cancer research and drug safety assessment [2,5,6].

Focusing on spheroids, various techniques have been used for their formation, with the same principle, whereby adhesive forces between cells are more prominent than cell–substrate adhesion [2,7]. Producing uniform and homogenous spheroids in terms of size, shape, and compactness level has proved to be challenging using conventional spheroid formation methods such as liquid overlay technique (LOT) [7,8], hanging drop method [9], spinner flasks [10], and stirred tanks [11]. Recent advances in microengineering and microfabrication aim to address this limitation by developing microfluidic cell culture systems to generate more uniform, reproducible spheroids by providing better control over the cellular microenvironment [12,13]. These devices are particularly useful for capturing small numbers of circulating tumor cells from patient liquid biopsies [14]. However, most of the developed platforms only rely on the design of the channels and cell-trapping chamber geometries to effectively direct cell aggregations [15,16,17,18]. Surface engineering has become more popular in the past decades due to the importance of substrate surface properties to alter cellular responses (e.g., cell adhesion) [19,20]. However, there remains ample room for surface optimization toward spheroid-on-a-chip technology. PDMS has a long history of use in microfluidic biochip platforms [12,21]. The hydrophobic nature of the PDMS [12], which is associated with the methyl groups present in the PDMS structure [22,23], leads to protein adsorption and consequently cell adhesion to the PDMS surface is problematic specifically in the cell culture-based platforms [12,21]. Therefore, the optimal surface engineering approach to render PDMS more hydrophilic remains to be determined for tumor-on-a-chip studies and drug screening [12,24,25,26].

We previously designed a PDMS-based tumor-on-a-chip model for capturing tumor cells and generating uniform spheroids [27]. We observed that 10% BSA can render PDMS surface hydrophilic and block adhesive properties, leading to higher cell–cell interactions compared to cell–substrate interactions. This resulted in homogenous spheroid production from MDA-MB-231 breast cancer cells conducive to future co-culture experiments and therapeutic screening. The objective of the present study was to expand the use of the microfluidic platform to multiple tumor cell types in an effort to broaden the scope of therapeutic screening. In this work, for the first time, the impact of surface modification of PDMS on the uniform and homogenous spheroid production from several tumor cell lines and the co-culture model of tumor–stromal cells has been studied.

## 2. Materials and Methodology

### 2.1. Fabrication and Surface Treatment of the Microfluidic Devices

#### 2.1.1. Fabrication of Microfluidic Biochip to Form Spheroids

**Figure 1:**
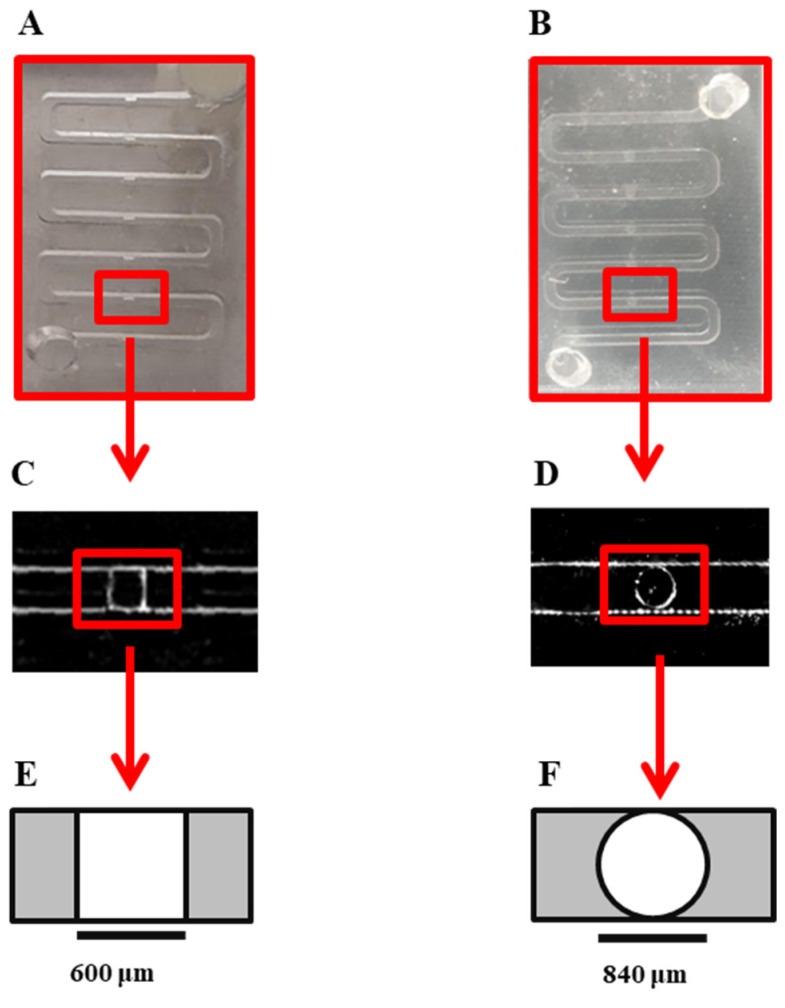
Microscopic image and schematic of the cube-shaped cell-trapping microwell: (**A**) Image of the channel in previous design with cube-shaped cell-trapping chamber. Each channel has a length of 78 mm with a cross-section of 600 μm × 600 μm with five cube-shaped cell-trapping chambers for cell sedimentation and spheroid formation. (**B**) Image of the modified design used in this work with cylindrical-shaped cell-trapping chambers. Each channel has a length of 109.2 mm with a cross-section of 840 μm × 840 μm with five cylindrical cell-trapping chambers for cell sedimentation and spheroid formation (**C**) Top view of microscopic image of cell-trapping chamber of the previous design; the size of the chamber is 600 μm × 600 μm and the height of the chamber is 540 μm. (**D**) Top view microscopic image of modified cylindrical-shaped cell-trapping chamber in the present study; the radius of the chamber is 840 μm and the height of the chamber is 756 μm. (**E**) Schematic top view of cube-shaped cell-trapping chamber in previous design. (**F**) Schematic top view of cylindrical-shaped cell-trapping chamber in the current design used in this work.

The general design of the channel is similar to our previous work, which was adapted from the design developed by Astolfi et al. [28]. In this work, the shape of the microwells was modified to the cylindrical form instead of the cube shape to prevent sticking the cells to the corners of the microwells and promote spheroid formation on the device (Figure 1). This device is composed of two PDMS layers. The bottom layer of the device consists of two open channels with 840 μm × 840 μm wide square cross-sections. Each channel contains five 840-μm-diameter circle-bottom microwells 756 μm in height. The top layer PDMS has 3-mm-diameter inlet and outlet holes bonded with Plasma Oxygen to the bottom layer as described before. 3D-printed molds (HTM 140 resin, EnvisionTEC GmbH, Gladbeck, Germany) were kindly printed by Prof. D. Juncker’s lab, Biomedical engineering department, McGill University, using a stereolithography-based printer (Perfactory MicroEDU, EnvisionTEC Inc., Gladbeck, Germany).

To obtain microfluidic devices from the 3D-printed mold, we made PDMS replicas by soft lithography. To prevent sticking of the PDMS to the resin mold, we pre-treated molds with a silicone spray (Ease Release 200^®^, Mann Formulated Products, Macungie, PA, USA) prior to replication of PDMS. To make PDMS replicas, as described before [27], elastomer base and curing agent (Sylgard 184 polydimethylsiloxane elastomer kit, Dow Corning, Midland, MI, USA) were mixed properly in a 10:1 ratio. The mixture was then degassed for about 1 h and poured into the 3D-printed mold while placed in a petri dish. PDMS replicas were cured for 2 h in a 65 °C oven and then peeled off from the mold. Due to the presence of the silicone spray residues, the first PDMS replicas from each mold were discarded and subsequent replicas were used to fabricate microfluidic devices. The two PDMS layers bonded together to form closed microfluidic devices following surface treatment with oxygen plasma at Prof. M. Wertherimer’s lab, Engineering Physics department, Polytechnique Montreal. An oxygen gas (air liquide, alphagaz 1, 99.999% purity) was set at a flow rate of 20 sccm (standard cubic centimeters) controlled by a mass flow controller (MKS 247c channel readout + MKS 1259B-00100RV (0–100 sccm). The pressure in the chamber (cylindrical with a 9″ diameter and 1.25″ height) was set at 600 mTorr (80 Pa) and fixed using a butterfly valve during the process. The plasma was initiated by radiofrequency plasma at 13.56 MHz (power source: ENI model HF-300+ impedance matching unit, plasma therm AMNS 3000-E) and the applied power was set at 20 Watts, with a plasma exposure time of 20 s.

#### 2.1.2. Surface Treatment of PDMS Biochip

Before introducing the cells to the devices, by using 99.9% ethanol (Sigma-Aldrich, St. Louis, MO, USA), microfluidic channels were sterilized and the air bubbles were removed from the channels. To reduce cell adhesion to the PDMS surfaces and promote the spheroid formation, we performed surface coating with BSA. BSA (Sigma-Aldrich) solutions in two different concentrations of 3% *w*/*v* and 10% *w*/*v* in sterile phosphate buffered saline (PBS, Sigma Aldrich) were prepared [27] and sterilized by filtration through a 0.2 μm filter (Millipore Sigma). The microfluidic channels were flushed out 3–4 times with the BSA solutions to remove the ethanol residual.

Then, by incubation of freshly prepared BSA solutions with biochips, their surfaces were modified to assess spheroid formation on-chip. One group of biochips was surface treated with 3% BSA, and another group of biochips was surface treated with 10% BSA to assess the impact of surface treatment on spheroid production. Only 10% BSA was used for co-culture model assays. All the devices were incubated overnight in the incubator (37 °C, 5% CO_2_, 95% ambient air) right after the cell seeding process.

#### 2.1.3. Cell Culture and Spheroid Formation On-Chip

Six different cell lines were used in this study: epithelial breast cancer cell line MDA-MB231 (Green-fluorescent protein, GFP), stromal connective tissue cells including IRM-90 mCherry Fibroblasts (Red-fluorescent protein, RFP), derivative subline of human prostate cancer LNCaP-derived C4-2B cells, human non-small lung adenocarcinoma cell line H1299, human lung carcinoma epithelial cell line A549, and human glioblastoma cell line U251. MDA-MB231, IRM-90, and C4-2B were kindly provided by the laboratory of Prof. M. Park at McGill University. H1299 and A549 were generously provided by the laboratory of Prof. M. Lavertu at Polytechnique Montreal. U251cell line was a generous gift from the laboratory of Dr. R. J. Diaz at The Montreal Neurological Institute-Hospital, McGill University. C4-2B, H1299, A549, and U251 cells were fluorescently labeled with cell-labeling solution (Vybrant Dio, V22886; Molecular Probes, Invitrogen, Thermofisher, Montreal, QC, Canada) in accordance with the manufacturer’s protocol.

H1299 and C4-2B cells were cultured in RPMI-1640 medium supplemented with 10% *v*/*v* fetal bovine serum (FBS) and 1% *v*/*v* penicillin/streptomycin (PS). All the other cell lines were cultured in high-glucose Dulbecco’s Modified Eagle’s Medium (DMEM) supplemented as above (all products from Gibco, Thermofisher, Montreal, QC, Canada). All the cell lines (less than 5 passages) were seeded in T-75 flasks (Corning Inc., New York, USA) at the density of 1.5 × 10^6^ cells per flask and placed in the incubator at 37 °C, 5% CO_2_ and 95% relative humidity (RH). After reaching 80–90% confluence, cells were washed two times with sterile phosphate buffered saline (PBS, Gibco, Thermofisher, Montreal, QC, Canada), and trypsinized with 0.25% Trypsin-EDTA solution (Gibco, Thermofisher, Montreal, QC, Canada). Then supplemented fresh culture medium (Gibco, Thermofisher) was added to the cell suspensions and centrifuged for 5 min at 1500 rpm to collect the cells. Prior to cell seeding, microfluidic channels were rinsed properly with sterile PBS (Gibco, Thermofisher) three times to clean the channels from the BSA residues, then the fluid within the channels was replaced with a fresh supplemented culture medium. Then, cell suspensions with the concentration of 1 × 10^6^ cells/mL were injected into the microfluidic channels inlets by using a micropipette (P200) and at the same time, 100 µL of the medium were removed from the channel’s outlet. Consequently, the cell suspension was pipetted into the outlet, and 100 µL of cell suspension inside the channel was removed from the inlet. To provide uniform and homogenous cell distribution across the channels, we repeated this process on both sides (inlet and outlet) of the channels three times. Following the cell seeding process, microfluidic devices were put in the incubator. The culture medium was refreshed every 24 h using a micropipette (P200).

### 2.2. On-Chip Observation of Spheroid Formation and Proliferation

Using Epifluorescence inverted microscope (Axio Observer.Z1, Zeiss, Oberkochen, Germany) and sCMOS camera (LaVision, Göttingen, Germany) and the objective lens EC Plan-Neofluar 5×/0.15, spheroid formation and proliferation were imaged directly through the thin PDMS layer for the duration of 7 days. The size of the spheroids, their compactness, and their spherical geometry was quantitated on day 1, 3, and 7 using Image J software.

### 2.3. Image J and Data Analysis

The images of the spheroids that were recorded as explained above were used for analysis with Image J (National Institute of Health, Maryland, PA, USA) to assess spheroid growth and proliferation. All images had a high resolution of more than 1900 dpi. The brightness level of fluorescent images was assessed by a digit value from zero to a maximum of 255. The average mean diameter of each spheroid can be written as D m = (D max + D min)/2 from 10 repeats. We reported the average size of the spheroids as mean diameter ± standard deviation. We defined the circularity of the spheroids by the Circularity = 100 × (D min/D max around a single sphere). Brightness level ratio is equal to BLR = (Brightness level/255) × 100. We used fluorescent signals as the function of cell proliferation in the spheroids. All Data have been reported as the mean ± SE of minimum of three independent tests. All error bars in figures indicate SE. One representative experiment is presented where the same trends were seen in multiple trials.

### 2.4. Assessment of Multicellular Spheroid Co-Culture Model On-Chip

We assessed spheroid morphology and proliferation in co-culture models. 3D tumor model consisting of a co-culture of A549 cells and IRM-90 cells (Ratio of 1:1, 1:2, 2:1) have been used to form hetero-type MCTSs in comparison with a monoculture of either A549 or IRM-90. The initial cell suspension had a concentration of 1 × 10^6^ cells/mL in all the experiments. Process of cell seeding and spheroid formation on-chip was repeated as described above for mono-type MCTSs.

### 2.5. Adhesion and Morphology Assays on Biochips Treated with 3% BSA

As a comparison and a control model for spheroid formation assays on PDMS biochips that were surface treated with 10% BSA, spheroid formation and cell adhesion were assessed on biochip treated with 3% BSA.

### 2.6. Statistical Analysis

All experiments were repeated in three independent biological replicates (*n* = 3) in order to evaluate the reproducibility of the data. For each experiment, a minimum of 10 spheroids was analyzed per condition. Statistical analyses were performed using Microsoft Excel 2016 (Version 1803, Build 9126.2259). All data are represented as mean ± standard error of the mean. One-way ANOVA was performed to assess statistical significance between means, with post hoc Tukey tests for comparison between means. A *p* ≤ 0.05 was considered statistically significant.

## 3. Results and Discussion

To generate compact and uniform spheroids, adhesive forces between cells and their substrate need to be weaker than the adhesive forces between the cells. Our previous work [27] demonstrated the impact of the surface properties of the PDMS in uniform spheroid formation on-chip. Our observations indicated that PDMS biochips surface modified with 10% BSA provide optimal cell-repellent properties characterized by greater surface wettability (moderate contact angle between 70° to 60°) when compared with the bare PDMS (contact angle ≥ 100°). The PDMS surface modification with 10% BSA has illustrated a desirable surface microstructure upon which MDA-MB 231 cells reproducibly do not tend to adhere. Hence, PDMS surface treatment with 10% BSA promoted cell self-aggregations and homogenous spheroid formation on-chip. On the other hand, the biochips that were surface treated with 3% BSA demonstrated lower surface wettability (contact angle ≥ 90°) when compared with 10% BSA, upon which MDA-MB 231 cells tend to adhere to the surface. The microstructure of PDMS surfaces treated with 3% BSA was less smooth with less surface coverage when compared with PDMS surfaces treated with the 10% BSA. Therefore, the quantity and quality of cellular self-aggregations and spheroid formation on devices drastically were reduced. We previously studied the impact of PDMS surface modifications on spheroid formation using MDA-MB 231 cells. Since cells’ adhesive interactions with the surfaces are complicated due to the different cells’ surface receptors and various surface properties [29,30,31], in the present study, we investigated the impact of our surface-optimized biochips treated with 10% BSA on the uniform and homogenous spheroid production from several cell lines.

### 3.1. Design and Fabrication of the Microfluidic Biochip

**Figure A1:**
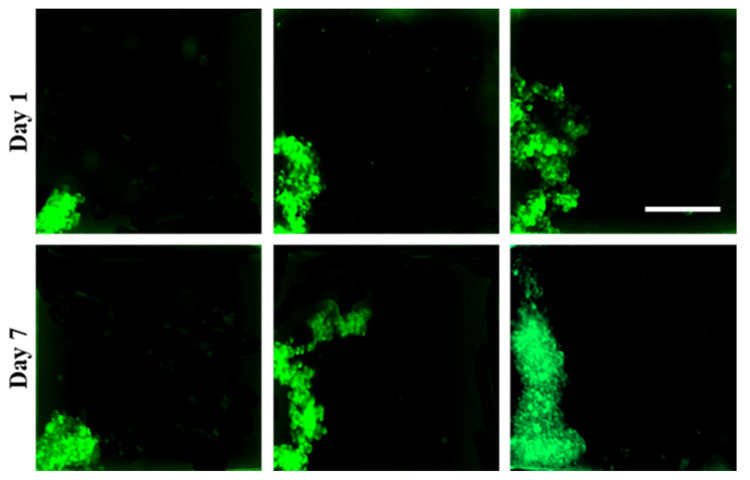
MDA-MB-231 cells stick to the corners of the wells.

The physicochemical properties of the microfluidic biochips play a crucial role in spheroid formation in devices [27]. In the present study, we optimized the design of the microwells to improve the chance of spheroid formation on our surface-treated device. We developed a design [27] adapted from Astolfi et al. [15,28]. We observed that the cube shape of the wells could increase the chance of cell adhesion to the corners of the cell-trapping microwells, and could affect the quality of spheroid formation by increasing the chance of producing lobular shapes and non-spherical cell aggregations (Figure A1). To overcome this limitation, in this work, we modified the form of the cell-trapping microwells to cylindrical shapes, which resulted in more effective cell aggregation and a more centered position of the resulting cell clusters: Figure 1.

### 3.2. Spheroid Formation on Model Surfaces Treated with 3% and 10% BSA

The uniform spheroid formation on surface-modified PDMS biochips was studied. We observed that biochips treated with 10% BSA solution boast a greater amount of spheroid formation on-chip and increased quality of spheroids for the various cell lines studied here, in a reproducible manner.

**Figure 2:**
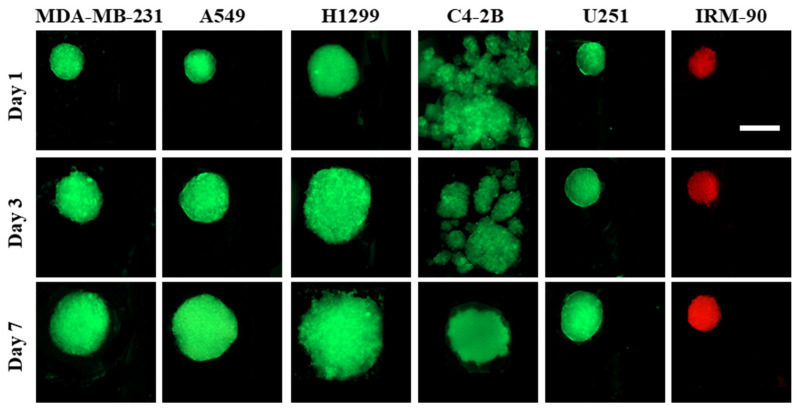
Spheroid formation on PDMS biochips pre-coated with 10% BSA. Fluorescent images of spheroids captured on days 1, 3, and 7 showed that each cell line had its own proliferation rate in the 3D model. All cell lines studied here were able to form spheroids during 7 days of culture. The red color is due to red-fluorescent protein (RFP) in IRM-90 cells and the green color is due to green-fluorescent protein (GFP) in MDA-MB-231 cells. For other cell lines, the green color is due to being fluorescently labeled with cell-labeling solution Vybrant Dio V22886. Scale bars: 200 μm.

The principle of spheroid formation is the same for all of the cell lines studied in this work. After micro-channels were filled with the cell suspension, cells began to sediment into the cylindrical microwells. When the surfaces of the channels were pre-treated with 10% BSA, cell–cell adhesive forces became dominant over cell–substrate adhesive forces. Hence, during the first 24 h of incubation, cell aggregations occurred for all the cell lines and the co-culture models, except for C4-2B cells, where spheroid formation was not observed during the first 72 h of culture. After 24 h, non-aggregated and aggregated cells inside the channels were rinsed with a fresh medium. During the second day of incubation (48 h post-seeding), proliferation and spheroid compaction were observed for all the cell lines and co-culture models, except for C4-2B cells. For C4-2B cells, spherical cell agglomerations were observed on day 3 (72 h) post-seeding. All the cell lines were successfully made spheroid on biochips pre-coated with 10% BSA and spheroid growth was assessed over a period of seven days (Figure 2).

**Figure 3:**
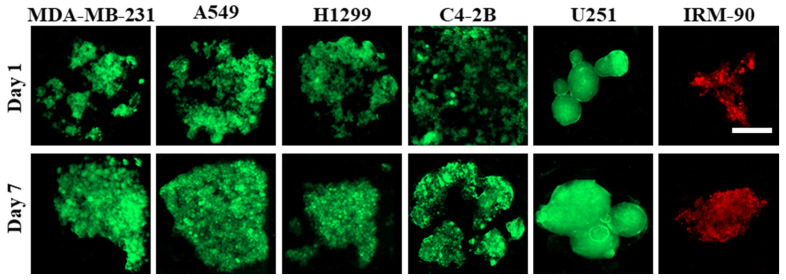
Culture of different cell lines on PDMS biochips pre-coated with 3% BSA. Cell adhesion pattern to the surface is different, and it depends on the cell type. In some cell lines, like C4-2B, cell–substrate interactions are dominant over cell–cell interactions; therefore, they cannot form compact cell clusters on surfaces treated with 3% BSA. On the other hand, some cell lines like U251 demonstrated greater cell–cell interactions when compared with cell–substrate interactions; therefore, this leads to the formation of more compact cell clusters. The red color is due to red-fluorescent protein (RFP) in IRM-90 cells and the green color is due to green-fluorescent protein (GFP) in MDA-MB-231 cells. For other cell lines, the green color is due to being fluorescently labeled with cell-labeling solution Vybrant Dio V22886. Scale bars: 200 μm.

Laminar flow in microfluidic channels provided protection of spheroids from the shear stress caused by exchange of medium, while the diffusion-based mass exchange is quick enough to effectively remove cellular waste and provide nutrition for spheroids [15,28,32]. We observed that the proliferation pattern was different in 3D for different cell lines, which leads to different sizes of spheroids on day 7 post-seeding, while the initial cell concentration is the same (1 × 10^6^ cells/mL) in all groups. As a control model, we studied the spheroid formation on biochips pre-coated with 3% BSA (Figure 3). When the cell suspension was introduced to the channels, due to the higher protein adsorption to the surface, cell–substrate interactions were greater than cell–cell interactions. This leads to cell adhesion to the surface, depending on the cell type, which can influence the quality of cells’ self-aggregations and spheroid production.

### 3.3. Spheroid Characterization

Routine monitoring of the spheroids was performed for 7 days. Fluorescent images of the spheroids were recorded directly from the thin PDMS layers. By using Image J software, morphometric parameters including minimum and maximum diameter, spheroid size, BLR, and circularity were obtained. Various parameters (e.g., flow rate, cell density during seeding, duration of cell seeding) modulate spheroid size [2] on the same design/size of channels/chambers of a microfluidic platform. Here, we studied the influence of cell type in the spheroid size, growth and spherical shape on our optimized design treated with 10% BSA.

**Figure 4:**
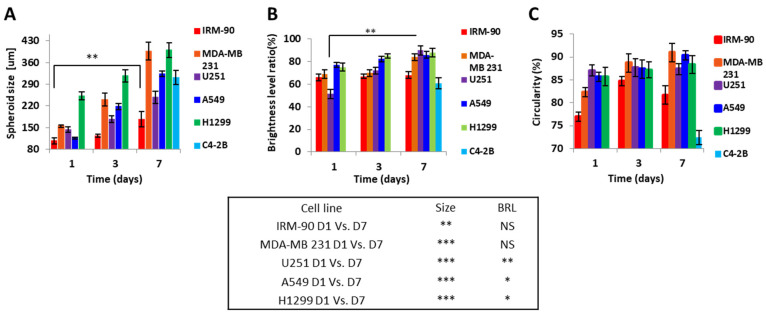
Proliferation and growth pattern for spheroid production on biochip pre-coated with 10% BSA. (**A**) Spheroid size over the seven days of culture. Spheroid size varied and was significantly increased on day 7 compared to day 1 for all cell lines (D1 vs. D7 ** *p* < 0.001 and *** *p* < 0.0001). C4-2B cell line was only able to produce spheroid after day 3 of culture, and we accordingly only saw the size on day 7. (**B**) BRL for U251, A549, and H1299 significantly increased on day 7 compared to day 1 (D1 vs. D7 * *p* < 0.05 and ** *p* < 0.001) and for IRM-90 and MDA-MB 231 varied non-significantly (NS = non-significant). (**C**) Circularity is representative of the spherical or circular shape of spheroids. Circularity increased non-significantly on day 7 compared to day 1 for all of the cell lines. C4-2B cell line only was able to produce spheroid after day 3 of culture, as previously mentioned. All assays were performed as triplicates per trial in three independent experiments. (Error bars represent ± SE, *n* = 3).

Figure 4 shows the seven-day growth pattern of the spheroids cultured on biochips pre-coated with 10% BSA.

Here, we observed that, contrary to 2D cultures, which grow exponentially, spheroids were characterized by an exponential growth phase followed by a decrease in proliferation with an increase in non-proliferation rate. According to our observations, the duration of each phase was dependent on the type of the cell line.

### 3.4. Study of Co-Culture Multicellular Spheroids

Spheroids can also be formed from a co-culture of tumor cells with stromal cells [2,3]. These co-culture models are advantageous for cancer research, as they better mimic the complexity of the tumor microenvironment [3,33].

**Figure 5:**
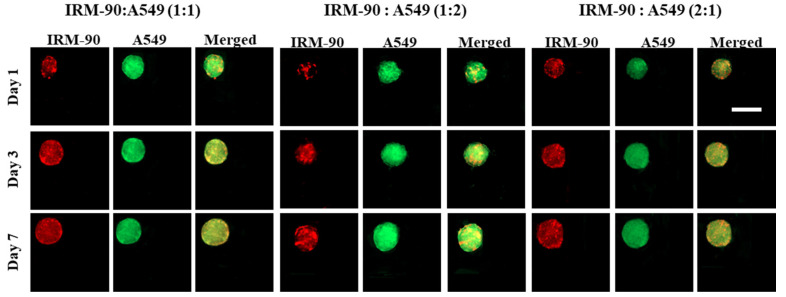
Image of co-culture A549 and IRM-90 cells with different ratios between the two cell lines. The total initial cell concentration was 1 × 10^6^ cells/mL for all experiments. Our observations demonstrated that there were no remarkable differences in terms of the growth pattern including the size and the shape of the spheroids by varying the ratio between tumor and stromal cells. The red color is due to red-fluorescent protein (RFP) in IRM-90 cells and the green color is due to being fluorescently labeled with cell-labeling solution Vybrant Dio V22886 for A549 cells. Scale bars: 200 μm.

We developed scaffold-free multicellular spheroids consisting of tumor cells (A549) and fibroblast (IRM-90) to provide the crosstalk between tumor and stroma cells. Figure 5 shows the images of the co-culture models for three ratios of tumor–stromal cells, including 1:1, 1:2, and 2:1, for a total number of 1 × 10^6^ cells/mL in each experiment.

**Figure 6:**
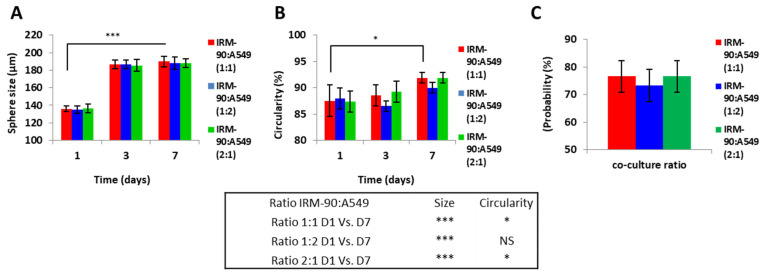
MCTSs were produced from different ratios of tumor (A549) and stromal (IRM-90) cells. (**A**) Spheroid size on days 1, 3, and 7 of culture. In all three ratios between tumor and stromal cells, the size of the spheroids significantly changed on day 7 when compared to day 1 (D1 vs. D7 *** *p* < 0.0001). However, spheroids from different ratios of tumor–stromal cells (1:1, 1:2 and 2:1) on day 7 did not have a statistically significant difference in size. (**B**) Circularity of spheroids for the stromal–tumor ratio of IRM-90:A549 1:1 and IRM-90:A549 2:1 was increased significantly on day 7 when compared to day 1 (D1 vs, D7 * *p* < 0.05) and for IRM-90:A549 1:2 was not statistically significant (non-significant = NS). However, spheroids from different ratios of tumor–stromal cells (1:1, 1:2, and 2:1) on day 7 did not have a statistically significant difference in terms of circularity. (**C**) Probability of spheroid formation on-chip. By changing the ratio of tumor and stromal cell probability, spheroid production did not change statistically significantly. The total number of cells is 1 × 10^6^ cells/mL for all three ratios. *p* < 0.05 is considered statistically significant. All assays were performed as triplicates per trial in three independent experiments. (Error bars represent ± SE, *n* = 3).

We studied the effect of changes in the ratio of tumor cells and stromal cells on the size and growth pattern of the co-culture models. In terms of spheroid size, circular shape, and the chance of spheroid formation on-chip, no significant changes were observed when changing the ratio between the tumor and stromal cells in this study (Figure 6).

According to the data presented in Figure 6, since remarkable differences in terms of size, geometry, and the chance of spheroid formation were not observed when changing the ratio between A549 and IRM-90 cells in our experiments, we chose the ratio of 1:1 between tumor cells (A549) and fibroblasts (IRM-90) for further experiments. In this regard, we studied the effect of the presence of the stromal cells in the tumor microenvironment on spheroid size, morphology, and chance of spheroid formation.

**Figure 7:**
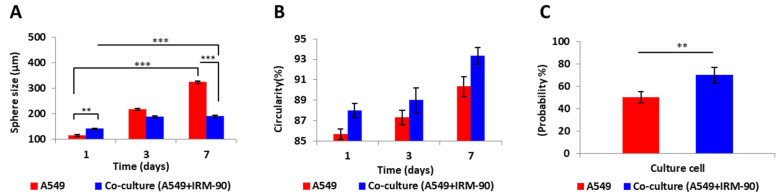
Comparing mono-type MCTSs produced from tumor cells (A549) with hetero-type MCTSs of tumor: stromal (A549:IRM-90, ratio 1:1) cells. (**A**) Changes in spheroid size over 7 days of culture. In both conditions (mono-type and hetero-type MCTSs), size of the spheroids on day 7 was significantly increased when compared to day 1 (D1 vs. D7 *** *p* < 0.0001). Spheroid size on day 1 was significantly different when comparing mono-type with hetero-type MCTSs (** *p* < 0.001). The size of mono-type MCTSs compared with hetero-type MCTSs on day 7 was significantly different (*** *p* < 0.0001). The size of spheroids in the co-culture model did not change significantly after day 3 of culture, while in mono-culture, spheroid size increased significantly on day 7 when compared to day 3 (D3 vs. D7 *** *p* < 0.0001). (**B**) Circularity of the spheroids was not significantly different in mono-type MCTSs when compared to the co-culture model. (**C**) Probability of spheroid formation on-chip is greater in the co-culture model when compared with the monotype MCTS model (** *p* < 0.001). The total number of cells is 1 × 10^6^ cells/mL for all three ratios. *p* < 0.05 is considered statistically significant. High-resolution images of 10 spheroids in each independent experiment have been considered for calculations per each condition. All assays were performed as triplicates per trial in three independent experiments. (Error bars represent ± SE, *n* = 3.).

Spheroid size on day 7 of culture was significantly different when comparing mono-type MCTSs with hetero-type MCTSs, which consist of stromal cells in their microenvironment (*** *p* < 0.0001). However, the circularity of the mono-type and hetero-type MCTSs did not show a statistically significant difference. Moreover, the chance of spheroid formation on the device by adding the stromal cells into the tumor microenvironment significantly increased (** *p* < 0.001) (Figure 7). We considered the images from spheroids having a ratio of 1:1 between the tumor and stromal cells as the co-culture model in our calculations.

It is proven that the presence of the stromal cells in the tumor microenvironment increases the physiological relevance of the spheroid models and influences the cellular responses to therapeutic agents [3,7]. Our observation demonstrated that the presence of the fibroblasts cells (IRM-90) in the tumor microenvironement provides dynamic interactions and strong adhesion between tumor cells, stromal cells, and their ECM. This can explain why we do not see a remarkable change in the size of the heterotype MCTSs after day three while they proliferate and become more compact with uniform and spherical shapes

## 4. Conclusions

Spheroid size and morphology influence the robustness of in vitro assays [2]. Therefore, the generation of homogenous spheroids in terms of size, compactness, and spherical morphology is important for reliable drug testing [2,4,7]. The relationship between surface properties and cell adhesion is complex, and it depends on various factors including but not limited to surface wettability, charge, morphology, roughness, and also to the type of the cells and cell’s surface receptors [34,35,36,37]. In this work, we used 10% BSA to develop surface-optimized biochips with having optimal cell repellent properties. We studied the effect of surface modification on the uniformity of spheroid production from several cell lines.

The microenvironment, including multicellularity, can drastically influence the formation of spheroids [3,38]. Recapitulating this microenvironment is highly important for assessing physiological responses to therapy [7]. We observed that cell behavior is deeply influenced by their cellular microenvironment and the surface properties of the substrate on which they grow.

Here, we conclude that surface treatment with 10% BSA is a simple, effective, and reproducible method for surface modification of PDMS-based biochips to promote the formation of scaffold-free spheroid models on-chip for various cell lines studied in this work. This platform holds the possibility to be used as a tool for the assessment of the efficacy of various therapeutic strategies in vitro. A fundamental understanding of interactions between cell-surface is particularly important for directed control of cell function for biomedical applications. Future work will explore capturing patient-derived circulating tumor cells from liquid biopsies, as well as assess the impact of spheroid formation from patient-derived solid-tumor and stromal cells. The continued research effort in the field of biomaterials and surface engineering in microfluidic platforms will help to create desirable surfaces for lab-on-a-chip and organ-on-a-chip applications.

## Data Availability

Not applicable.

## Acronyms

BRL	Brightness level ratio
BSA	Bovine serum albumin
DMEM	Dulbecco’s Modified Eagle’s Medium
ECM	Extra cellular matrix
LOT	Liquid overlay technique
MCTS	Multi cellular tumor spheroid
PBS	Phosphate buffered saline
PDMS	Polydimethylsiloxane
PS	Penicilline/Streptomycin
PVA	Polyvinyl alcohol
RH	Relative humidity
SD	Standard deviation
SE	Standard error
3D	Three-dimensional
2D	Two-dimensional
WCA	Water contact angle

## Appendix A

Cells have more chance to stick to the corners of the cube-shaped cell-trapping microwells design of our microfluidic device, therefore in the present study, the shape of the cell-trapping micro wells have modified to cylindrical shapes to decrease this phenomenon.

Cells sometimes tend to attach to the corners of the cube-shaped cell-trapping microwells even though the surfaces were pre-coated with 10% BSA. This could lead to a decrease in the chance of spheroid formation on chip by increasing the possibility of forming the lobular or any non-spherical cell agglomerations. Figure A1 represent different forms of cell aggregation produced in different cube-shaped cell-trapping chambers in our old design. MDA-MB-231-GFP cells are pictured in this image. The green color is due to green-fluorescent protein (GFP) in MDA-MB-231 cells. Scale bars: 200 μm.
